# *In Vitro* Effect of Mouthrinses on the Microhardness of Three Different Nanohybrid Composite Resins

**DOI:** 10.1155/2023/9161639

**Published:** 2023-11-07

**Authors:** Jhonn Luis Bernaldo-Faustino, Julissa Amparo Dulanto-Vargas, Kilder Maynor Carranza-Samanez

**Affiliations:** Research Group in Dental Sciences, School of Dentistry, Universidad Científica del Sur, Lima, Peru

## Abstract

**Introduction:**

Daily use of different mouthrinses at home or in the dental office can alter the properties of resin hardness. The present study aimed to compare Vickers microhardness (VHN) *in vitro* of nanohybrid composite resins (NCRs) immersed in mouthrinses.

**Materials and Methods:**

In total, 120 discs (10 ⨯ 2 mm) were prepared from three NCR shade A2 (*n* = 40/group) with Filtek^TM^ Z350 XT (Z350XT), Tetric EvoCeram (TEC), and Polofil NHT (PNHT). The specimens were incubated in artificial saliva (37°C ⨯ 24 hr). Specimens were distributed into four mouthrinses (*n* = 10/group) of artificial saliva (control), chlorhexidine (CHX 0.12%, pH 5.6), cetylpyridine chloride (CPC 0.075%, pH 5.84), and CHX 0.12% + CPC 0.05% (pH 6.2) (2 times/day, 2' ⨯ 12 hr). The VHN (300 gf/10”) was measured after 24 hr, 14, and 21 days. Data were analyzed by three-way analysis of variance, followed by post hoc Tukey analysis at 0.05 level of significance.

**Results:**

The results revealed a global effect of the interaction of the mouthrinses ⨯ time between NRC evaluated (*P*=0.001). Baseline VHN in PNHT and Z350XT was higher than TEC. Within each group of NCR, VHN of CHX + CPC > other mouthrinses (PNHT/14 days; Z350XT/21 days), and >CPC (Z350XT/14 days). In mouthrinses-intragroups, VHN of PNHT and Z350XT decreased at 14 days (CHX, CPC) and was maintained over time in NCR (CHX + CPC). VHN-TEC was similar among groups.

**Conclusion:**

Microhardness showed differences due to the interaction of the type of NCR, the composition of the mouthrinses, and time. VHN decreased after 14 days and was more affected in composite resins with lower filler content and in mouthrinses with a lower pH.

## 1. Introduction

Toothbrushing is the main conventional method of oral hygiene to prevent oral diseases. It is well known that this cleaning requires other mechanical procedures, such as dental flossing [1]. However, situations can arise that prevent dental biofilm control, and people resort to the use of oral rinses, which are usually available without a dental prescription. Recent reviews of certain rinses for adjunctive use recognize the beneficial effect on plaque reduction, as in the case of chlorhexidine (CHX) and cetylpyridine chloride (CPC) after four weeks of use. However, some side effects, such as extrinsic tooth staining, calculus, and taste alteration, are also reported [1, 2].

Oral rinses are formulated from essential oils, salts, preservatives, and water and have a slightly acidic pH [3, 4]. They may contain various concentrations of alcohol, although “free alcohol” rinses containing broad-spectrum antimicrobial agents such as cationic biguanide (CHX), cationic quaternary ammonium (CPC), and the combination of both (CHX + CPC) are increasingly being marketed for common or office use [3, 5]. Due to the COVID-19 pandemic, the virucidal benefits of these mouthrinses in dental procedures have been studied [6, 7].

Composite resin has been used for more than 60 years and is the universally accepted restorative material. The demand for high functional esthetics has led to a rapid modification of this material, mainly focusing on its monomeric components, including particle size, shape, and loading [8]. Modern “nanohybrid” composite resins are characterized by the presence of silica fillers of different sizes, such as nano (20–75 nm) and submicron (≤1 *μ*m) particles [9]. Their frequent use is due to their versatility in restoring both anterior and posterior teeth [10]. The variety of filler sizes among different commercial brands produces similar stiffness and strength, but this does not define their properties when considering other elements such as content, shape, and distribution [11].

The resin matrix may degrade in the presence of alcohol due to the hydrophilicity of the polymer [12]. In addition, a low pH can cause loss of ions by catalyzing ester groups of dimethacrylate monomers (Bis-GMA, Bis-EMA, UDMA, TEGDMA). This effect can cause changes in composite resin properties, such as hardness [13–16], roughness [12, 17, 18], and surface morphology [14]. Although the use of antimicrobial mouthwashes is a justified complement to brushing to maintain periodontal health and control anticariogenic activity, the detrimental effects on composite resin restorations must be considered [4]. Since the daily use of mouthrinses at home and the selection of dental composite in clinical practice can alter the chemical properties, reducing longevity and durability [12, 19]. These effects are important to study because they play a crucial role in reducing additional treatments and improve patient satisfaction [9].

The degradation of composites resin exposed to chemical solutions has been extensively studied; however, few studies have been carried out on nanohybrid composite resins (NCRs) and alcohol-free mouthrinses. Therefore, the aim of the present study was to evaluate the change in Vickers microhardness (VHN) of NCRs (Filtek Z350XT, Tetric EvoCeram, and Polofil NHT) immersed in mouthrinses of different compositions (CHX, CPC, CHX + CPC) using an *in vitro* procedure. As a null hypothesis, it was proposed that none of the mouthrinses would have a significant effect on reducing the VHN of the three NCRs at 21 days postimmersion in an *in vitro* model.

## 2. Materials and Methods

This study had an *in vitro* experimental design and was approved by the Research Ethics Committee at the Universidad Científica del Sur (no. 140-CIEI-CIENTÍFICA-2022). A total of 120 composite resin disc samples were prepared and distributed into 12 groups of NCR (Filtek Z350XT, Tetric EvoCeram, and Polofil NHT) and four mouthrinses (artificial saliva control, CHX 0.12%, CPC 0.075%, and CHX 0.12% + CPC 0.05%) (*n* = 10, per group). The discs were prepared according to the manufacturer's instructions (ISO standard 4049), with standard dimensions (diameter 10 mm; thickness 2 mm, margin of error ≤1 mm). The characteristics of the materials are shown in Table 1.

The sample size was calculated using the paired means comparison formula of the Epidat Program (version 4.2), based on the study of CHX, CPC, and artificial saliva by Jyothi *et al*. [13] using the difference of the two closest means (∆_pre- vs. postimmersion_ = 1.5) found in the experimental group of CPC at 0.075% (∆SD ≈ 1.5), 95% confidence interval, and 80% power; resulting in 10 samples per subgroup. The samples were randomly assigned to three experimental groups of mouthrinses and one control group of artificial saliva for each type of composite resin.

The composite resin discs were prepared according to the ISO technical standard 4049 [13, 17, 20]. The stainless-steel mold was lubricated with petroleum jelly to facilitate sample removal. The mold was placed on and under two twin slide plates (22 mm ⨯ 22 mm) separated by celluloid matrix tape to prevent the formation of an oxygen-inhibited surface layer that reduces hardness. The composite resin was introduced in homogeneous 1 mm increments to a slight excess using a spatula. Finger pressure was applied to both plates for 20 s until the excess composite resin was removed and a smooth, homogeneous surface was obtained. Each side of the disc was automatically light cured for 20 s with an LED lamp (1000–1200 mW/cm^2^; Woodpecker Led F) calibrated with a radiometer (intensity ≥1100 mW/cm^2^, spectral range 460–480 nm) at 1 mm from the surface (glass slide thickness) [14, 16]. The composite resins were polished with coarse, medium, fine, and extra-fine Sof-Lex discs (3M ESPE, USA) under running water.

The samples were randomly distributed in pill containers labeled 1–120 according to the type of mouthrinses and composite resins. They were kept in artificial saliva in an incubator for 24 hr at 37 ± 3°C to simulate the oral environment. The artificial saliva had the following composition: NaCl (0.084 g), KCl (0.120 g), CaCl_2_·2H_2_O (0.015 g), MgCl_2_·6H_2_O (0.005 g), sodium carboxymethylcellulose (0.375 g), propylene glycol (4 g), methylparaben (0.1 g), propylparaben (0.01 g), and purified water (100 ml) (Salival®, Lusa, Laboratorios Unidos S.A., Perú).

The immersion cycle experiment was the same for the three mouthrinses: prerinse after 24 hr of immersion in artificial saliva and drying with absorbent paper; two minutes of immersion twice a day every 12 hr; postrinse with distilled water after each immersion for a period of 21 days. The remainder of the time, they were immersed in artificial saliva and kept in an incubator at a mean temperature of 37°C, while the sample of artificial saliva was kept in the same place all the time. The mouthwash and artificial saliva solutions were changed every 24 hr. To prevent any chemical changes in the solutions, the samples were kept in dark containers, and pH values were controlled.

**Figure 1:**
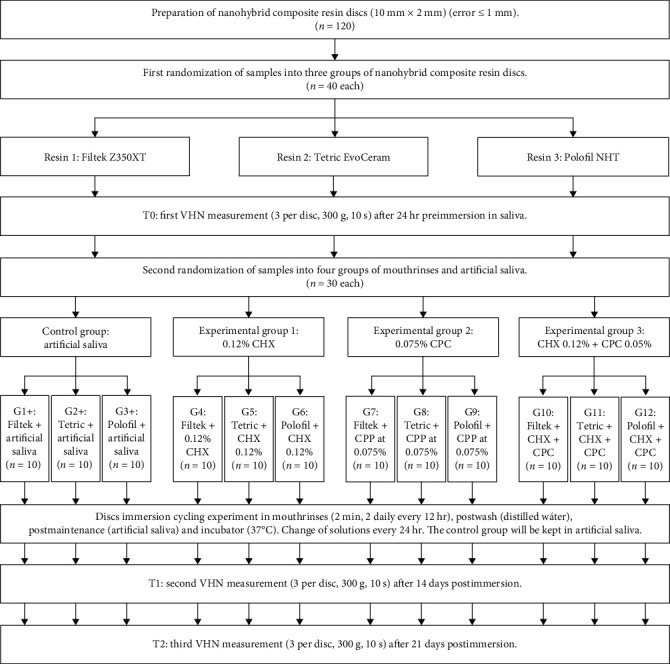
Flowchart of the experiment in nanohybrid composite resins.

Microhardness was measured by the Vickers method in all the samples in ascending order of the labeling at 24 hr of artificial saliva immersion (baseline preimmersion = T0) and at 14 (T1) and 21 days (T2) postimmersion. The mean VHN measurements of each surface of the composite resin disc were performed by three randomly performed diamond indentations (2 *μ*m in diameter). A microhardness tester (LG HV 1000; High Technology Laboratory Certificate S.A.C.) was used at 30 N (300 gf) for 10 s^20^. The formula used to calculate VHN was the applied load divided by the surface area (diagonals) of the indentation (mm^2^) multiplied by a constant (1.854), expressed in kgf. A description of the procedure is shown in the study flow chart (Figure 1).

The analyses were performed using Jamovi software version 2.3.17 (2021). The VHN was described in terms of mean and standard deviation (SD). Three-way analysis of variance (ANOVA) (repeated measures time (factor 1), mouthrinses (factor 2), and composite resins (factor 3)) with post hoc Tukey analysis (*P* < 0.05) were used according to data distribution.

## 3. Results

Significantly strong baseline VHN values were observed with Polofil NHT (84.3 ± 5.33) and Filtek Z350XT (78.4 ± 5.22) compared to Tetric EvoCeram (43.9 ± 2.43) (*P* < 0.001). Within the composite resin group, the VHN of CHX + CPC was higher than that of other solutions in Polofil NHT (14 days) and Filtek Z350XT (21 days) and higher than CPC in Filtek Z350XT (14 days) (*P* < 0.001) and was similar to that of the other solutions in Tetric EvoCeram (*P* ≥ 0.05). Within the immersion group, the VHN of Polofil NHT and Filtek Z350XT decreased at 14 days (CHX and CPC: *P* < 0.001) and 21 days (CHX, CPC and artificial saliva: *P* < 0.05), while it was maintained over time in all composite resins (CHX + CPC) and in Tetric EvoCeram (all solutions) (*P* ≥ 0.05) (Table 2). Statistically significant differences were found in VHN for the factors time, mouthrinses, and composite resins when analyzed separately (*P* < 0.001) and by interaction (*P* < 0.001) (Table 3).

**Figure 2:**
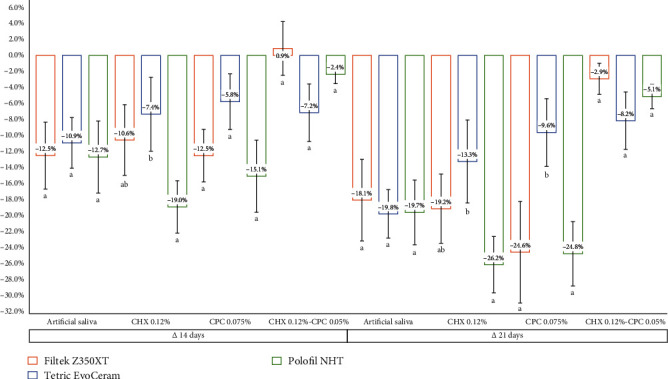
Comparison of microhardness variation among groups of nanohybrid composite resins for each mouthrinse (mean and standard error values evaluated by ANOVA with post hoc Tukey analysis are shown. Different lower-case letters indicate significant differences by rows, and different lower-case letters indicate significant differences by resins in the same mouthrinse/time).

The mouthrinse × composite resin interaction showed that the VHN of Filtek Z350XT and Polofil NHT was higher when immersed in CHX + CPC compared to other mouthrinses (*P* < 0.001 and *P* < 0.05, respectively), being similar in all solution groups for Tetric EvoCeram (*P* < 0.05). The VHN values of the immersion in CHX-CPC were higher with Filtek Z350XT and Polofil NHT compared to Tetric EvoCeram, while in the other mouthrinse groups, they were higher to lower with Filtek Z350XT > Polofil NHT > Tetric EvoCeram (*P* < 0.001). For the mouthrinse × time interaction, VHN was statistically equal among mouthrinse groups at baseline (*P* > 0.05) and higher at 14 and 21 days with CHX-CPC compared to other mouthrinses (*P* < 0.001). Intragroup VHN significantly decreased at 14 days for all mouthrinses except CHX-CPC, which show a reduction in VHN at 21 days (*P* < 0.05). Finally, in the composite resin × time interaction, VHN showed differences among NCR at baseline (Filtek Z350XT > Polofil NHT > Tetric EvoCeram) (*P* < 0.001) and was higher at 14 and 21 days with Filtek Z350XT and Polofil NHT versus Tetric EvoCeram (*P* < 0.001). The intragroup VHN significantly decreased between time points for all composite resins (baseline > 14 days > 21 days) (*P* < 0.05) (Table 4). There was a greater decrease in VHN with Polofil NHT compared to Tetric EvoCeram immersed in CHX after 14 days and with Polofil NHT and Filtek Z350XT compared to Tetric EvoCeram immersed in CPC after 21 days (*P* < 0.001) (Figure 2).

## 4. Discussion

Composite resin is the most widely accepted restorative material and has undergone modifications that differentiate its filler, matrix, and interaction characteristics. The properties of NCR could counteract local factors of oral rinses that could reduce the hardness of their surface and decrease the quality of oral health [21].

Mouthwash research is widespread; however, we found limited evidence on the long-term effects of oral antiseptics of different formulations on the properties of new technology resins. It is essential to understand whether NCRs are altered by chemical solutions since this could indirectly affect their efficiency of resistance to fracture, wear, and chewing. Although microhardness cannot measure the direct change in the composition of the resins before mouthwashes, it does reflect changes in hardness [8, 10–12, 19, 21, 22].

Determination of surface VHN is a conventional noninvasive technique frequently used to evaluate the physical properties of biomaterials such as resins [22, 23]. Greater resistance of the composite to indentation represents opposition to the influence of other structures providing greater durability over time and a lower risk of replacement due to failure [8–10]. Increasing hybrid filler content in the NCR microstructure would increase wear resistance and decrease aqueous absorption (saliva, beverages, acids, etc.) [19, 21].

The present study aimed to determine the *in vitro* effect of mouthrinses such as CHX 0.12%, CPC 0.075%, and the combination of the two (CHX 0.12% + CPC 0.05%) on the VHN of three different NCR at 14 and 21 days postimmersion. According to our analysis, a significant reduction in VHN was observed with respect to the combinations of mouthrinses, with the test confirming that there are no subsets among the microhardness of the composite resins.

In studies on the interaction of mouthrinses, resins, and measurement time, a reduction in VHN was observed in hybrid [14, 15] and nanofilled [13] composites resins. The NCR is a more recent innovation among resin-based compounds, developed with greater complexity in the interaction of organic and inorganic compounds to improve their optical properties [8, 10, 11]. Despite these improvements, it is important to evaluate their resistance to the interaction of adverse factors such as those evaluated in the present study. Despite the lack of previous studies that agree on the multiple interactions of types of manufacturing, mouthrinses, and measuring time, our results were consistent with the findings reported in other studies.

Previous research found that NCR showed less VHN involvement than hybrids when exposed to acidic liquids (pH ≤ 3.2) after 7 days [24] and 15 days [23]. Another study found less VHN involvement in Z350 XT resins, especially when exposed to CHX at 0.12 (pH = 6.07) compared to other classifications of resin fillers and mouthrinses with a lower pH (<4.1) after 7 days [25].

Regarding studies on the interaction of VHN with resin and time related to roughness conditions, higher resistance to indentation is reported in NCR [5], leading to higher durability and lower risk of replacement due to failure [8, 10], justifying the mechanical performance observed by Polofil NHT. The surface VHN then suggests a procedural advantage over a noninvasive technique that allows estimating the physical properties of biomaterials [22], since indirect analysis of wear and aqueous absorption can support a scientifically based decision. Thus, the determination of the highest reduction of VHN *in vitro* by the Polofil NHT resin immersion (−26.2%) in CHX at 0.12% can be considered the best alternative.

In studies on the interaction of mouthrinses and time on VHN, it was found that mouthrinses for adjunctive use, with or without the presence of alcohol, altered microhardness [16] or roughness [4, 17]. Mouthwash containing CHX and CPC are referred to recommended for its good antimicrobial properties that make it suitable for regular cleaning use at home and for surgical intervention, examination, and/or treatment in the office. On the other hand, due to the COVID-19 pandemic, in recent years, there has been a growing interest among health personnel in its incorporation as a routine protocol. Its use could reduce the viral load of the drops or aerosols generated during the dental visit, according to recent randomized clinical trials [26, 27].

Another result of interest was the interaction of VHN on composite resin in relation to time, with differences in NCR generally after 14 days of immersion, regardless of NCR manufacture and rinsing. The importance of this evaluation is to demonstrate that although mouthwashes used in dental clinics can be quite useful for daily use in patients, their prolonged use can ultimately degrade resin restorations. In addition to this, other adverse effects have been described in the literature, such as dry mouth, elimination of certain healthy bacteria, pigmentation, or taste disturbances [1–3, 5]. A control of the time of use must be prescribed and, in turn, monitored by professionals.

The findings of this study are consistent with those mentioned above. This could be explained in that a greater volume of the filler and a smaller particle size of composite resins based on nanohybrid compounds compared to hybrid compounds provide greater efficiency in the response to degradation of the polymer matrix, especially with low pH values [23, 25, 28]. Likewise, an acidic environment caused by interaction with alcohol-based mouthwashes can hydrolyze ester radicals into monomer-like compounds. Nonetheless, NCR has standard compounds with the substitution of phenolic groups (Bis-GMA and UDMA, and TEGDMA) and a greater surface interaction between their fillers, with both characteristics generating greater stability to water (less reabsorption), especially for counteracting chemical damage by mouthrinses with low pH values (acidity), higher alcohol content or a longer interaction with their use [12, 15, 24, 25, 28].

Regarding the methodological scope of this study, its *in vitro* nature may be modified by oral factors such as food and saliva. Although the necessary measures were taken to reduce the risks, isolating, and carefully using each element, the restorations of the studied elements may vary the expected results. However, there are limitations inherent to a simulated environment compared to a real (clinical) environment in which patients may present differences in mouthwash exposure times, different pH according to the type of mouthrinses, different capacities of saliva properties and temperature of the oral cavity, NCR with different compositions and different areas of NCR that are exposed to immersion. Therefore, it is recommended to carry out *in vivo* studies that determine a greater number of factors related to NCR degradation, including other physical measurements such as surface roughness and morphology.

## 5. Conclusions

Within the limitations of the present study, microhardness was found to decrease according to the individual and collective interaction of time, type of NCR, and the components of mouthrinses. Lower microhardness values were found after 14 days and were more affected in composite resins with lower filler content and in mouthrinses with a lower pH.

## Figures and Tables

**Table 1:** Description of the materials of the experiment.

Material	Composition	Manufacturer	Batch
*Rinses*			

Periogard	Chlorhexidine digluconate at 0.12%; water; glycerin; PEG-40; flavoring and sodium saccharin (alcohol free; pH 5.6)	Colgate Palmolive Ind.®	R1020

Colgate Plax	Cetylpyridine chloride at 0.075%; sodium fluoride 0.05% (225 ppm fluoride); water; glycerin; sorbitol; propylene glycol; poloxamer 407, potassium sorbate, menthol, and sodium saccharin (alcohol-free; pH 5.84)	Colgate Palmolive Ind.®	1040

Perio Aid	Chlorhexidine digluconate at 0.12%; cetylpyridine chloride at 0.05%; water; glycerin; xylitol; saccharin sodium; acesulfame potassium; neohesperidin (alcohol free; pH 6.2)	Dentaid	S1119

*Nanohybrid resins*			

Filtek Z350XT	Bis-GMA, Bis- EMA, UDMA y TEGDMA. Filler loading is 72.5% by weight with a combination of nonagglomerated/nonaggregated 20 nm silica filler; nonagglomerated/nonaggregated 4–11 nm zirconia filler	3M ESPE®	NE41882

Tetric EvoCeram	Bis-GMA, UDMA, Ethoxylated Bis-EMA. The filler loading is 82%–83% by weight with barium aluminum silicate glass with two particle sizes, ytterbium trifluoride, and mixed oxide	Ivoclar Vivadent®	Z02X74

Polofil NHT	TEGDMA, UDMA, BisGMA. The filler loading is 88.7% with glass silicate, silica, camphor quinone, dabe, bht, silica, ferric oxide, titanium oxide, benzotriazole, and methylphenol	Voco®	2114441

**Table 2:** Comparison of microhardness of nanohybrid composite resins according to mouthrinses and time.

Mouthrinses	Nanohybrid composite resins (media ± SD)								
	Filtek Z350XT			Tetric EvoCeram			Polofil NHT		
	Baseline	14 days	21 days	Baseline	14 days	21 days	Baseline	14 days	21 days
Artificial saliva	80.91 ± 5.62^Aa^	70.83 ± 7.69^ABb^	66.30 ± 8.18^Bb^	45.63 ± 2.24^Aa^	40.59 ± 2.18^Aab^	36.56 ± 2.36^Ab^	80.61 ± 4.39^Aa^	70.16 ± 4.09^Bb^	64.63 ± 4.62^Bc^
CHX al 0.12%	78.87 ± 6.50^Aa^	70.25 ± 4.73^ABb^	63.41 ± 3.37^BCc^	43.53 ± 2.95^Aa^	40.21 ± 2.72^Aa^	37.61 ± 2.75^Aa^	85.71 ± 0.92^Aa^	69.48 ± 4.91^Bb^	63.26 ± 4.87^Bc^
CPC al 0.075%	76.11 ± 3.86^Aa^	66.51 ± 4.21^Bb^	57.16 ± 6.56^Cc^	42.88 ± 2.39^Aa^	40.34 ± 2.33^Aa^	38.63 ± 1.88^Aa^	83.08 ± 6.32^Aa^	70.46 ± 7.90^Bb^	62.38 ± 6.40^Bc^
CHX 0.12% + CPC 0.05%	77.78 ± 4.00^Aa^	78.29 ± 1.83^Aa^	75.44 ± 3.15^Aa^	43.65 ± 1.24^Aa^	40.50 ± 2.31^Aa^	40.06 ± 2.35^Aa^	87.64 ± 5.64^Aa^	85.56 ± 5.71^Aa^	83.06 ± 4.34^Aa^

*Note*: Different capital letters indicate significant differences by columns. Different lower-case letters indicate significant differences by rows (separate composite resins).

**Table 3:** Interaction of variables mouthrinses, time, and nanohybrid composite resins.

Variables	df	MSE	F	*P*	*η* ^2^
Time	2	4018.5	399.12	<0.001	0.067
Mouthrinse	3	1184.6	28.68	<0.001	0.030
Composite resin	2	43478.5	1052.74	<0.001	0.728
Time × mouthrinse	6	226.8	22.53	<0.001	0.011
Time × composite resin	4	288.8	28.68	<0.001	0.010
Mouthrinse × composite resin	6	357.4	8.65	<0.001	0.018
Time × mouthrinse × composite resin	12	54.2	5.38	<0.001	0.005

^*∗*^*Note*: Interaction of variables. Type 3 sum of squares. Repeated measures ANOVA with post hoc Tukey analysis.

**Table 4:** Comparison of microhardness according to mouthrinse × composite resin, mouthrinse × time, and composite resin × time interaction.

Variables	Artificial saliva	CHX 0.12%	CPC 0.075%	CHX 0.12%–CPC 0.05%
Filtek Z350XT	72.68 ± 9.35^Aab^	70.85 ± 8.06^Abc^	66.59 ± 9.24^Ac^	77.17 ± 3.27^Ba^
Tetric EvoCeram	40.93 ± 4.36^Ba^	40.45 ± 3.66^Ba^	40.61 ± 2.78^Ba^	41.40 ± 2.55^Ca^
Polofil NHT	71.80 ± 7.95^Ab^	72.82 ± 10.38^Ab^	71.97 ± 10.93^Ab^	85.42 ± 5.43^Aa^
	Artificial saliva	CHX 0.12%	CPC 0.075%	CHX 0.12%-CPC 0.05%
Baseline microhardness	69.05 ± 17.35^Aa^	69.37 ± 19.22^Aa^	67.37 ± 18.36^Aa^	69.69 ± 19.57^Aa^
Microhardness 14 days	60.52 ± 15.19^Bb^	59.98 ± 14.80^Bb^	59.10 ± 14.54^Bb^	68.12 ± 20.41^ABa^
Microhardness 21 days	55.83 ± 14.89^Cb^	54.76 ± 12.86^Cb^	52.72 ± 11.60^Cb^	66.19 ± 19.33^Ba^
	Filtek Z350XT	Tetric EvoCeram	Polofil NHT	
Baseline microhardness	78.42 ± 5.22^Ab^	43.92 ± 2.43^Ac^	84.26 ± 5.33^Aa^	
Microhardness 14 days	71.47 ± 6.51^Ba^	40.41 ± 2.31^Bb^	73.92 ± 8.82^Ba^	
Microhardness 21 days	65.58 ± 8.65^Ca^	38.21 ± 2.61^Cb^	68.33 ± 9.95^Ca^	

*Note*: Different capital letters indicate significant differences by columns. Different lower-case letters indicate significant differences by rows. Repeated measures ANOVA with post hoc Tukey analysis.

## Data Availability

Data are available upon request.
